# Comparative evaluation of antimicrobial efficacy of three herbal irrigants in reducing intracanal *E. faecalis* populations: An *in vitro* study

**DOI:** 10.4317/jced.52339

**Published:** 2016-07-01

**Authors:** Alpa Gupta-Wadhwa, Jitesh Wadhwa, Jigyasa Duhan

**Affiliations:** 1 Senior resident. Department of Conservative Dentistry & Endodontics, Post Graduate Institute of Dental Sciences, Rohtak; 2 Senior lecturer. Department of Orthodontics, Inderprastha Dental College, Sahibabad; 3 Professor. Department of Conservative Dentistry & Endodontics, Post Graduate Institute of Dental Sciences, Rohtak

## Abstract

**Background:**

The present study aimed to evaluate the intracanal bacterial reduction promoted by chemomechanical preparation using three different herbal extracts named *Ocimum sanctum (OS), Cinnamomum zeylanicum (CZ), Syzygium aromaticum (SA) against Enterococcus faecalis*.

**Material and Methods:**

Root canals from extracted teeth were contaminated with *Enterococcus faecalis* ATCC 29212 for 7 days and then randomly distributed into 3 experimental groups of 10 teeth each: which includes conventional irrigation with OS, CZ and SA. The control groups included 5 teeth each consisting of NaOCl (positive control) and distilled water (negative control). Samples taken before and after chemomechanical procedures were cultured, and the colony-forming units (CFUs) were counted. Bacterial identification was performed using Polymerase chain reaction technique. The statistical analyses were performed with various tests.

**Results:**

Reduction in the intracanal bacterial populations was highly significant for all the experimental groups. CZ and SA showed 80 to 85% intracanal bacterial reduction while *O. Sanctum* revealed only 70 to 75 % reduction. NaOCl showed 96 to 100 % bacterial reduction on the other hand distilled water showed very minimal bacterial reduction i.e 10 to 16%.

**Conclusions:**

*Cinnamomum zeylanicum, Syzygium aromaticum* and *Ocimum sanctum* showed intracanal bacterial reduction against *Enterococcus faecalis*. The 3 experimental groups were less effective in terms of intracanal bacterial reduction as compare to NaOCl but more effective than distilled water.

** Key words:**Antimicrobial activity, Cinnamomum zeylanicum, Enterococcus faecalis, Ocimum sanctum, Syzygium aromaticum, herbal extracts.

## Introduction

A large number of bacterial species play a major role in the development of pulp and periapical diseases (1-3). The privileged anatomic localization, allows bacterial entrenchment inside the root canal system and these bacteria are beyond the reach of host defense system (4). The dominant species which is associated with secondary infection is *Enterococcus faecalis* (*E. faecalis*) (5) and the range of its prevalence in persistent endodontic infections is 24% to 77% (6).

To eliminate intracanal bacterial populations completely or their minimum reduction that is compatible with periradicular tissue healing is one of the major microbiological goal of the chemomechanical preparation (4). Therefore, the use of irrigating solutions with strong antimicrobial activity is an essential adjunct to mechanical preparation in order to further reduce micro-organisms.

The excellent antimicrobial property of sodium hypochlorite (NaOCl) against planktonic (7) and biofilm (8,9) forms makes it an ideal root canal irrigant. But on the other side, it has several limitations such as tissue toxicity, allergic potential and disagreeable smell and taste (10). So the requirement of irrigants with low toxicity but high antimicrobial efficiency is the need of the hour in dentistry.

Bacteria persisting after chemomechanical procedures at levels detectable by culturing technique might jeopardize treatment prognosis (11,12). Therefore, efforts should be made to search for chemomechanical protocols that predictably promote negative cultures (4).

In the preliminary study, the extracts of *Ocimum sanctum* (*O. sanctum*), *Cinnamomum zeylanicum* (*C. zeylanicum*), *Syzygium aromaticum* (*S. aromaticum*) showed antimicrobial effects against *E. faecalis* both in planktonic and biofilm forms (13). With agar diffusion test, the study concluded that *O. Sanctum* had the least bacterial growth inhibition. In biofilm susceptibility assay (BSA) on cellulose nitrate membrane, NaOCl was associated with complete bacterial inhibition after contact of 2 minutes, while 10% *C. zeylanicum*, 10% *S. aromaticum* and 40% *O. sanctum* showed cessation of growth after 12, 12 and 24 hours, respectively. The results of BSA on tooth model were similar except for *O. Sanctum*, which was not included in the model (13). Till date, to the best of our knowledge no study has been conducted to reduce the intracanal bacterial population using herbal extracts as irrigants.

The previous study was performed using the plant extracts in immersion form (13). However the fact that mechanical instrumentation accompanied by irrigation is considered as the most essential component to achieve the primary objective of root canal treatment cannot be ignored (14-17). Moreover, the previous study was not performed inside the instrumented root canal of whole tooth model using these plant extracts as an irrigant (13). Hence, the present study aimed to compare the antibacterial efficacy of *S. aromaticum*, *O. sanctum* and *C. zeylanicum* extracts as irrigants with NaOCl and distilled water against *E. faecalis* (ATCC 29212) inside the instrumented root canals.

In addition, the present study aimed to evaluate reduction in intra-canal *E. faecalis* populations using minimum and clinically feasible irrigation time. Further, the effect of these plant extracts inside the whole tooth model was considered to evaluate the clinical utility of the extracts as irrigant and to simulate the conditions of root canal environment.

The culturing technique is preferred in the present study as it is widely available method, it allows quantification of all major viable cultivable microorganisms in a sample (18). At the same time, the accurate identification of bacteria with aberrant phenotypic behavior along with high sensitivity, specificity is provided by molecular biological methods like Polymerase chain reaction (PCR) technique (19). Hence PCR technique was used in the study for accurate identification of bacteria.

## Material and Methods

The medicinal plants used for the experimental purpose were *S. aromaticum*, *O. sanctum* and *C. zeylanicum* at their minimum bactericidal concentration (MBC). In line with the results of previous study (13), the MBC of *O. sanctum* was 200 mg/500µL of 30% dimethyl sulfoxide (DMSO) i.e. 40%, *C. zeylanicum* was 50mg/500µL of 30% DMSO i.e. 10% and *S. aromaticum* was 50mg/500µL of 30% DMSO i.e. 10%. The method of preparation of above mentioned plant extracts with their major constituents has been explained in the earlier study (Gupta *et al.* 2013). A pure culture of the test strain, *E. faecalis* ATCC 29212 (Department of Microbiology, PGIMS, Rohtak, Haryana, India) was prepared in sterile brain heart infusion (BHI) broth (HiMedia Laboratory Private Limited, Mumbai, Maharashtra, India) and adjusted spectrophotometrically (Shimadzu UV 2400 PC, Tokyo, Japan) to an optical density of 560 nm corresponding to match the turbidity of McFarland 0.5 scale [1.5x108 colony forming unit per millilitre (CFU mL-1)] and incubated at 37°C for 24 h.

-Specimen preparation and contamination

The study was approved by the institutional review board of Pandit Bhagwat Dayal Sharma Institute of Health Sciences, Rohtak, Haryana, India for collection and use of extracted teeth. The teeth were collected from the department of Oral and Maxillofacial Surgery, Rohtak, Haryana with the informed consent of the donor. A total of 40 maxillary and mandibular single rooted non-carious, extracted human teeth with fully developed apices were included in the study. Presence of a single canal was determined by radiographs taken in both the mesiodistal and buccolingual directions. The selected teeth ranged from 21 to 25 mm in length with intact clinical crowns. The teeth were cleaned to remove superficial debris, calculus and tissue tags and were stored in normal saline in order to prevent dehydration before use. Conventional access cavities were prepared using round burs and Endo-Z burs (Dentsply Maillefer, Ballaigues, Switzerland). The working length was established by introducing a K- type file of size 10 or 15 (Dentsply Maillifer, Ballaigues, Switzerland) in the canal until its tip was visualized at the apical foramen. In order to standardize the apical constriction size, the root canals were instrumented at the apical foramen with K-type file up to size 25 (Dentsply Maillifer, Ballaigues, Switzerland) in reaming action under irrigation with distilled water. Afterwards, the foramen was sealed with super glue to prevent bacterial leakage. To make handling easier, the teeth were mounted vertically up to the cervical region in blocks made up of silicon impression material (Dentsply, Aquasil, Germany). The blocks containing the teeth were sterilized in autoclave for 20 min at 121°C and 15 psi. Each root canal was completely filled with the *E. faecalis* suspension by using sterile 1 mL insulin syringe (Collateral Medical Private Limited, Mumbai, Maharashtra, India) without overflowing. Sterile K-type files of size 15 (Dentsply, Maillifer, Ballaigues, Switzerland) was used to deliver the bacterial suspension to the entire root canal length. Blocks were then placed inside a metallic box & incubated at 37°C for 7 days. Fresh culture medium was added to the canal at 1, 4 and 6 day after the initial inoculum under laminar flow hood (Bionics Scientific Technologies Private Limited, Sindhora Kalan, New Delhi, India). After 7 days of experimental contamination the bacterial identification was performed by PCR method during sampling procedure.

-Testing procedure

The teeth were randomly divided into three experimental groups of plant extracts with 10 teeth each in group A: *O. sanctum* extract; group B: *S. aromaticum* extract; group C: *C. zeylanicum* extract and two control group of five teeth each in group D: 3% NaOCl (Neodent, Karol Bagh, New Delhi, India) (as positive control) and group E: Distilled water (Parenteral Drugs Limited, Baddi, Himachal Pradesh, India) (as negative control). Root canals were instrumented using the same standardized procedure for all the groups.

The coronal and middle segments of the canal were prepared with rotary Protaper Universal instruments (ProTaper, Dentsply Maillefer, Ballaigues, Switzerland) and finally F2 and F3 were used to prepare the canal up to the working length.

-Irrigation protocol

Canals were initially irrigated with 2 mL of experimental extract for 30 s. After each instrument used, the canal was irrigated with 2 mL of tested extract by using a 30-gauge needle (Septodont, Henry Schein, Australia) adapted to a disposable plastic syringe.The needle was placed up to 3mm short of the working length. After the last instrument was used, experimental extract was left undisturbed for 60 s & then the finally irrigated with 2 mL of 3% NaOCl followed by 5 mL of 17% ethylene diamine tetra acetic acid (EDTA) (Neodent, Karol Bagh, New Delhi, India) for 1min & again with 2 mL of experimental extract. Overall, 20 mL of irrigant was used per canal for approximately 6 min 30 s. The same protocol was followed for both the control groups i.e distilled water (negative control) and 3% NaOCl (positive control).

-Sampling procedures

The root canals were sampled before (S1) and after (S2) chemomechanical procedure. Canals were filled with 0.9% sterile saline solution and the S1 sample was taken by the sequential use of 4 paper points placed to the working length. Each paper point remained in the canal for 1 min. To avoid contamination, all the procedures were performed in laminar flow hood. For positive control group, the root canal was flushed with 1 mL of 10 % sodium thiosulfate (Giant Bio Care Agro Private Limited, Vadodara, Gujarat, India) and, thereafter, with saline to neutralize the effect of NaOCl before taking S2 sample. The volume of sodium thiosulfate before S2 (1 mL) was included in the total volume calculation for positive control group. For S2 sampling of experimental groups, each root canal was rinsed with saline after final irrigation with extracts and a H-type file of size 30 or 40 (Dentsply Maillifer, Ballaigues, Switzerland) was used to vigorously file the dentinal walls. Thereafter, samples were taken using paper points as described for S1. Two paper points were used for culturing and other two for PCR for bacterial identification. Paper points were transferred to tubes containing 1 mL of BHI broth for culture. The test tubes were incubated for 24 h at 37°C. For both S1 and S2 samples, presence of broth turbidity was indicative of bacteria remaining in root canal. To confirm the presence of *E. faecalis* in the turbid broth, a 10 fold serially diluted sample was taken from these tubes & was cultured on blood agar plates and incubated for 24 h. Presence of colonies was indicative of *E.faecalis* growth. The CFUs grown were counted and then transformed into actual counts based on the known dilution factors.

-Detection of *E.faecalis* by PCR

The other two paper points were collected in Tris-EDTA buffer (HiMedia Laboratory Private Limited, Mumbai, Maharashtra, India) and frozen at -20°C to perform PCR technique. The bacterial DNA was isolated and detected as described by Al- Ahmad *et al.* (20). 100 µL of each specimen was centrifuged at 12,000g for 5 min and the resulting pellet was boiled in 100µL lysis buffer (10 mmol/L Tris-HCl buffer, 1 mmol/L EDTA, 1% Triton X-100, and pH 8.0) (HiMedia Laboratory Private Limited, Mumbai, Maharashtra, India) for 10 min. After centrifugation at 12,000g for 10 min, the supernatant containing DNA was used for PCR. The oligonucleotide species-specific primers for *E. faecalis* detection were 5’-GTT TAT GCC GCA TGG CAT AAGAG-3’ (forward primer) and 5’-CCG TCAGGG GAC GTT CAG-3’(reverse primer), producing a PCR amplicon of 310 base pairs (Sigma Aldrich Company, St. Louis, USA). PCR amplification was performed in a reaction volume of 50 µL, consisting of 0.8 µmol/L concentration of each primer, 5 µL of 10X PCR buffer, 2 mmol/L MgCl2, 2 U of *Taq*DNA polymerase and 0.2 mmol/L concentration of each deoxyribonucleoside triphosphate (HiMedia Laboratory Private Limited, Mumbai, Maharashtra, India). PCR amplification was performed in a DNA thermocycler (Gene AMP PCR 9700, Applied Biosystem). Cycling parameters included an initial denaturation step at 95°C for 2 min, followed by 35 cycles of a denaturation step at 95°C for 30 s, a primer annealing step at 60°C for 1 min, an extension step at 72°C for 1 min, and a final step of 72°C for 2 min. The results of PCR amplification were examined by electrophoresis in 1.5% agarose gel (Sigma Aldrich Company, St. Louis, USA). DNA was stained with ethidium bromide and visualized under short wavelength UV light (Fotodyne, Hartland, USA). Positive reaction was determined by the presence of band of expected size (310 base pairs).

-Statistical analyses

S1 data were submitted to the normality test of Kolmogorov-Smirnov D. Because it revealed a normal distribution for all groups (experimental and control), the Student t test was used to compare the initial infection between groups. The Mann-Whitney test was used for intragroup analyses (reduction from S1 to S2). Percent reduction in the number of CFUs was calculated on the basis of quantitative data obtained from S1 and S2. The Kruskal-Wallis test was used for the intergroup comparative analysis of S2 data. Dunn multiple comparison test was used to isolate the differences between the experimental and the control groups. The significance level was set at *p* <.05.

## Results

-Microbial culture

 Table 1 reveals the mean, median, range, and mean percent reduction of CFUs observed for all groups. Intragroup analyses revealed that in all groups (experimental and control), the reduction in the number of CFUs from S1 to S2 was highly significant (*p* < .05). Intergroup analysis of S1 samples revealed no significant difference, indicating that the method of experimental contamination was capable of providing a homogeneous and reliable baseline of bacterial load. Consequently, data from S2 could be used for direct intergroup comparisons, which were carried out by using the nonparametric Kruskal-Wallis test. We found that there is highly significant difference in terms of bacterial reduction between all the groups after irrigation except between *C. zeylanicum* and *S. aromaticum*. In terms of mean percentage reduction in bacterial load, *C. zeylanicum* and *S. aromaticum* were at the same level. NaOCl showed 96 to 100 % bacterial reduction followed by *C. zeylanicum* and *S. aromaticum* (80 to 85%) while *O. Sanctum* (70 to 75%) revealed minimum reduction. Distilled water showed very minimal bacterial reduction (10 to 16%). All experimental groups showed less percentage reduction of *E. faecalis* populations than the positive control group i.e NaOCl.

**Table 1:**
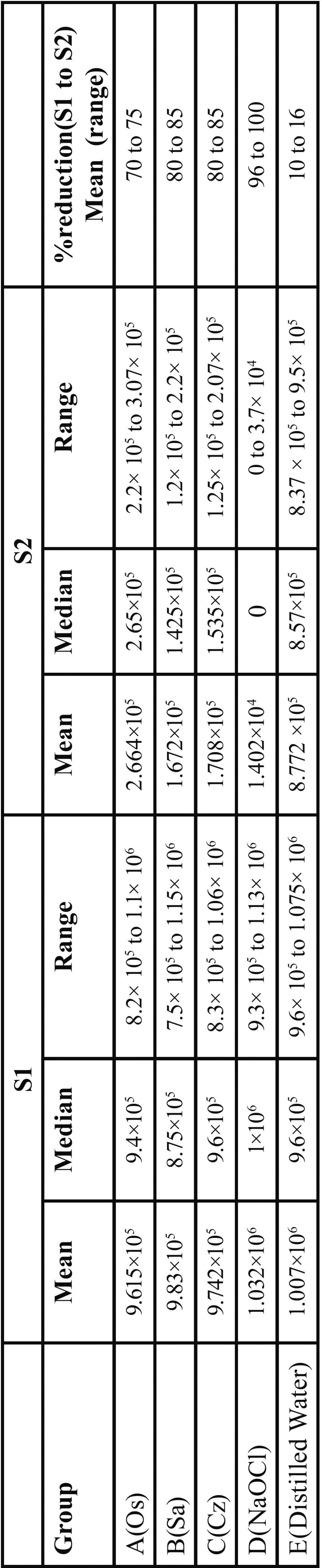
Bacterial count of *E. faecalis* (CFUs) before and after chemomechanical preparation using experimental and control groups as irrigant.

-Detection of *E. faecalis* by PCR

**Figure 1:**
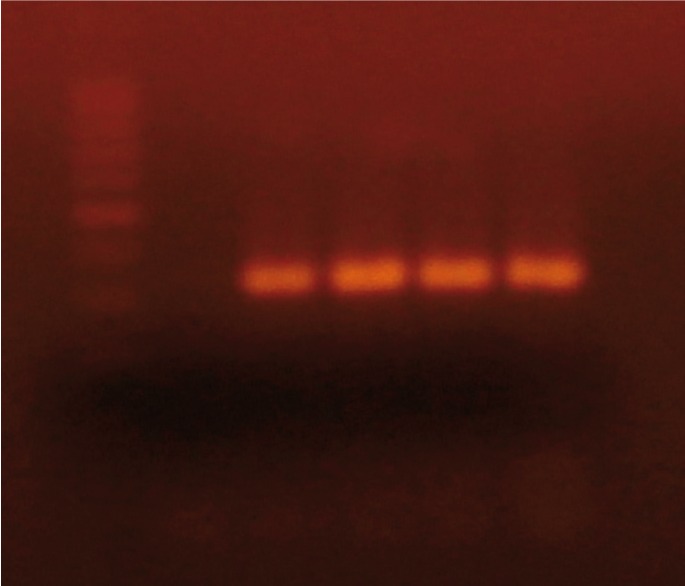
Positive reaction (indicating the presence of *E. faecalis* in the sample) was determined by the presence of band of expected size (310 base pairs).

Positive reaction (indicating the presence of *E. faecalis* in the sample) was determined by the presence of band of expected size (Fig. 1) by all the experimental and control groups both during S1 and S2. In two samples of NaOCl group, no band was observed after irrigation which indicates absence of *E. faecalis* in the sample.

## Discussion

Medicinal plants are a source of great economic value all over the world (21). Herbal medicine is still the mainstay of about 75-80% of the whole population and the major part of traditional therapy involves the use of plant extracts and their active constituents (22).

A preliminary study to evaluate the antimicrobial ability of *O. sanctum*, *C. zeylanicum*, *S. aromaticum* plant extracts against *E. faecalis* has already been performed explaining their major constituents along with their mechanism of action (13). The previous study concluded that the extracts of *O. sanctum*, *C. zeylanicum*, *S. aromaticum* showed antimicrobial activity against *E. faecalis* both in planktonic and biofilm forms, though NaOCl was found to be superior among all the groups.

Culture technique was used to detect *E. faecalis* inside the root canal because it is one of the most reliable methods to detect viable bacteria, particularly when samples are taken immediately after antibacterial treatment in which viability might not be ascertained by most culture-independent methods (23,24).

The results, while using experimental plant extracts inside the tooth model, revealed significant bacterial reduction with a particular irrigation time period. The antimicrobial efficacy of *O. sanctum* was low as compare to other two plant extracts, which is in line with our previous preliminary study. It may be attributed to lower concentration of active constituents.

The results shown by 3% NaOCl group in terms of bacterial reduction of present study with culture method were similar to earlier studies (25-27). It has been revealed by the studies that instrumentation and irrigation with NaOCl is not sufficient to render root canals free of cultivable bacteria. Even with varied concentration of NaOCl about 40%-60% of the canals still contain cultivable bacteria after chemomechanical preparation (24,28-31). The reason behind this is the design and rigidity of the endodontic instrument that allow them to act only in the main canal which might be insufficient to reach other areas of the root canal system, including irregularities, lateral canals, isthmuses, apical deltas, and dentinal tubules (24,29-31).

Two teeth with NaOCl came out with no bacterial count with culture method and also negative results with PCR technique. It is salient to point out that attainment of culture/PCR - negative cases does not always indicate the bacteria free canal. In such cases, bacteria might be in regions inaccessible to sampling procedure or they might be in the canal in levels below the sensitivity of the culture method (30).

In the detection of *E. faecalis* PCR is more sensitive than culturing regardless of type of sample and sampling site (32,33). The higher sensitivity of the PCR method could be partially attributed to the fact that it targets free floating DNA and DNA from non-viable, viable but nonculturable (VBNC) in addition to culturable viable cells. In contrast, culture-based identification methods require viable cells (34). *E. faecalis* has been found at consistently higher percentages (67-77%) with PCR detection method as compared to culturing (24-70%) (5). The method provides detection of bacterial species directly in a sample, but the limitation is that the technique is expensive and moreover most assays reveal qualitative or semiquantative results (23).

The present *in vitro* study highlights the antimicrobial ability of these plant extracts as irrigants against *E. faecalis* inside the instrumented root canals. The limitation of this study is the use of paper points for sampling, due to which bacteria located in other regions of the entire root canal system (including ramifications, dentinal tubules, isthmi, irregularities, and some untouched areas of the main canal) can remain unnoticed (35). Trials investigating the antimicrobial effect of these plant extract as irrigant in the clinical set up are required after evaluating their cytotoxicity for endodontic purpose. Further, more research work is required to conclude the endodontic application of these plant extracts as root canal irrigant or medicament.

## Conclusions

The extracts of *O. sanctum*, *C. zeylanicum*, *S. aromaticum* showed significant reduction in *E. faecalis* populations inside the instrumented root canals as irrigants, though NaOCl was found to be superior among all the groups.
